# Gains in body mass and body water in pregnancy and relationships to birth weight of offspring in rural and urban Pune, India

**DOI:** 10.1017/jns.2022.75

**Published:** 2022-09-09

**Authors:** Elaine C. Rush, Lindsay D. Plank, Himangi Lubree, Dattatray S. Bhat, Anjali Ganpule, Chittaranjan S. Yajnik

**Affiliations:** 1 Faculty of Health and Environmental Sciences, Auckland University of Technology, Auckland, New Zealand; 2 Department of Surgery, Faculty of Medical and Health Sciences, University of Auckland, Auckland, New Zealand; 3 Diabetes Unit, KEM Hospital Research Centre, Rasta Peth, Pune, India

**Keywords:** Bioimpedance spectroscopy, Birth weight, Body water by deuterium dilution, Nutrition transition, Pregnancy, BIS, bioimpedance spectroscopy, D_2_O, deuterated water, GWG, gestational weight gain, KEMH, King Edward Memorial Hospital, SLI, standard of living index, TBW, total body water.

## Abstract

Maternal size, weight gain in pregnancy, fetal gender, environment and gestational age are known determinants of birth weight. It is not clear which component of maternal weight or gained weight during pregnancy influences birth weight. We evaluated the association of maternal total body water measured by the deuterium dilution technique (TBW-D_2_O) at 17 and 34 weeks of gestation with birth weight. A secondary aim was to examine the utility of bioimpedance spectroscopy (BIS) to determine total body water (TBW-BIS) in pregnancy. At 17 and 34 weeks of pregnancy, ninety-nine women (fifty-one rural and forty-eight urban) from Pune, India had measurements of body weight, TBW-D_2_O, TBW-BIS and offspring birth weight. At 17 weeks of gestation, average weights for rural and urban women were 45⋅5 ± 4⋅8 (sd) and 50⋅7 ± 7⋅8 kg (*P* < 0⋅0001), respectively. Maternal weight gains over the subsequent 17 weeks for rural and urban women were 6⋅0 ± 2⋅2 and 7⋅5 ± 2⋅8 kg (*P* = 0⋅003) and water gains were 4⋅0 ± 2⋅4 and 4⋅8 ± 2⋅8 kg (*P* = 0⋅092), respectively. In both rural and urban women, birth weight was positively, and independently, associated with gestation and parity. Only for rural women, between 17 and 34 weeks, was an increase in dry mass (weight minus TBW-D_2_O) or a *decrease* in TBW-D_2_O as a percentage of total weight associated with a higher birth weight. At both 17 and 34 weeks, TBW-BIS increasingly underestimated TBW-D_2_O as the water space increased. Differences in body composition during pregnancy between rural and urban environments and possible impacts of nutrition transition on maternal body composition and fetal growth were demonstrated.

## Introduction

The birth weight of babies in India is relatively low compared with European^(1)^. Unlike developed countries,^(2)^ there are no specific guidelines for gestational weight gain (GWG) in India,^(1)^ but it is estimated that weight gain during pregnancy is on average 7 kg^(3)^ and the average birth weight of rural babies is 2⋅6 kg^(4)^. The proportion of people living in rural India in 2020 was 68 % as compared with people in rural areas having a lower standard of living than those who are in urban (not slum) areas. Rates of migration from rural to urban areas occur in relation to work, marriage, business and education and are increasing (https://censusindia.gov.in/) alongside the nutrition transition with changes in diet, physical activity and socio-economic status^(5)^. The city of Pune in Maharashtra, India is undergoing high population growth in large part due to migration from rural areas^(6)^. In 2016, the national prevalence of low birth weight (<2⋅5 kg) was 16⋅4 %, with less low birth weight in urban areas at 15⋅7 % compared with rural at 16⋅7 %^(7)^. One fifth of global births each year are in India. In an extensive literature search, we could find no publications that investigated, in India or other developing countries, maternal body composition and water changes in pregnancy.

Maternal size, weight gain in pregnancy, fetal gender and gestational age are known determinants of birth weight^(7,8)^. It is not clear which component of maternal weight or weight gained during pregnancy influences fetal size at birth or if this is different by the area of residence. It is challenging to measure changes in body composition during pregnancy as the maternal and fetal depots cannot easily be differentiated^(9)^ and the relative contributions to body mass that include amniotic fluid, uterine muscle, breast size and increase of extracellular fluids with gestation. The basic assumptions about the constancy of hydration of the fat-free mass in the two-component model are not valid in pregnancy^(10)^. A measure of fat-free mass by, for example, dual-energy x-ray absorptiometry (aside from the safety issue in pregnancy) would, therefore, not provide an accurate estimate of total body water (TBW). Hence, the measurement of TBW, usually the largest component of maternal weight, by the deuterated water (D_2_O) dilution method provides, at the time of measurement, an accurate measure of maternal total body water,^(11)^ and the proportion of body water that makes up total maternal weight can be derived.

Total body water, when measured by deuterium dilution, has been reported to increase by 5–8 kg during gestation, while total mass of the pregnant mother can increase between 12 and 16 kg^(9,12)^. These values of water and weight change are derived from urban women living in developed countries with pre-pregnancy weights more than 60 kg.

We hypothesised that an increase in maternal body water measured by deuterium dilution would be positively associated with an increase in birth weight, but that the increases in body mass would differ according to the rural or urban environment. A secondary aim was to examine the utility of bioimpedance spectroscopy (BIS) to determine TBW at 17 and 34 weeks of gestation and the changes over this period.

## Methods

Women with normal pregnancies registered at the rural primary health care centre at Vadu near Pune and at the antenatal clinic of King Edward Memorial Hospital (KEMH), Pune, Maharashtra, India were invited to participate in this cohort study. Inclusion criteria included normal singleton pregnancies booked before 16 weeks of gestation and consent to follow the study protocol entailing three visits during pregnancy: 17, 26 and 34 weeks of gestation are clinically relevant for the detection of anomalies, gestational diabetes mellitus and pre-eclampsia, respectively. Women were excluded if there was a congenital anomaly in the foetus or if there was a high risk of elective delivery. The study protocol was approved by the ethics committee of the KEMH Research Centre, Pune and by the local community leaders in the three study areas. Each subject signed informed consent.

Pregnant mothers had measurements of anthropometry and BIS at 17, 26 and 34 weeks of gestation at the Diabetes Research Centre, KEMH, Pune. Gestation was determined from the first day of the last menstrual period and modified if dating sonography varied beyond 2 weeks.

### Anthropometry

Trained observers performed all measurements. Maternal height was measured to the nearest 0⋅1 cm using a wall-fixed stadiometer (CMS Instruments, London, UK) and the body weight was recorded to the nearest 0⋅05 kg using a portable digital scale (Conveigh, Electronic Inst. Pvt. Ltd, Mumbai, India). After birth baby and mother measurements were completed mostly within 24 h and always within 72 h. Birth weight was measured to the nearest 1 g using a digital scale (Ishida, ATCO Pvt. Ltd, Mumbai, India), and after trimming of the umbilical cord and membranes, placental weight was recorded to the nearest 1 g with a digital scale (Ishida, ATCO Pvt. Ltd, Mumbai, India). Crown-heel length was measured to the nearest 0⋅1 cm using a portable Pedobaby Babymeter (ETS J.M.B., Brussels, Belgium).

After at least 5 min rest in the supine position, blood pressure was measured on the right arm using a digital monitor (Model UA 767PC, A&D Instruments Ltd, Abingdon, UK). Two readings were recorded and the second reading was used for analysis.

### Total body water by bioimpedance spectroscopy

Supine, hand to foot bioimpedance measurements were recorded in the morning after the bladder had been emptied, after fasting overnight and after resting 5 min in a supine position using a MultiScan 5000 device (Bodystat Ltd, Isle of Man, UK). TBW-BIS was estimated from measurements taken over 5–1000 kHz using a tetrapolar electrode arrangement and the manufacturer's Cole-Cole model-fitting software.

### Total body water by deuterium dilution

Total body water was measured by D_2_O^(13)^ at 17 and 34 weeks of gestation. Participants reported in the evening before the study and at 20.00 h received a standard dinner and then rested for 5 h. About 01.00 h, participants provided a basal urine sample. They then drank 75 mg/kg body weight of deuterated water (Europa Scientific, Crewe, UK) from a sterile plastic container with the aid of a straw. The dose was weighed to three decimal places on an Afcoset (FX-400) electronic balance (Mumbai, India). This was followed by 3 g/kg body weight of plain water using the same straw and the container to ensure that the complete dose was ingested. Urine samples were collected at the fifth and sixth hours after drinking the deuterated water. Body weight measured after the last urine sample was used for all calculations.

Urine samples were frozen (at −70°C) until transportation to St John's Hospital, Bangalore, India for further analysis. Deuterium enrichment was analysed by zinc reduction followed by dual-inlet mass spectroscopy (Europa Scientific, Crewe, UK)^(14)^. Each sample was analysed in duplicate, and the mean value was used for calculation. Repeated analysis for natural background samples gave a coefficient of variation (CV, delta *v*. Standard Mean Ocean Water, SMOW) equal to 0⋅02 %. With high enrichment samples using IAEA standard no. 302 (enrichment equal to 500 *v*. SMOW), CV was equal to 0⋅22 %. The sample dose of deuterated water was also analysed to ascertain its enrichment and included in the calculation of the deuterium dilution space. It is agreed that deuterium over estimates total body water by 4 % related to the exchange of hydrogen with other molecules,^(15)^ so the space was divided by 1⋅04 to derive total body water.

Based on the study by Butte that showed that in sixty-three women living in the United States, birth weight was correlated positively with gestational gain in TBW measured by deuterium dilution (*r* 0⋅37, *P* = 0⋅006), we calculated that forty-nine women were required from both the rural and urban environment to achieve an *α* value of 0⋅05 and power of 80 %.

### Statistical analysis

Descriptive data are reported as mean and standard deviation or numbers and percentages as appropriate. Differences between rural and urban maternal characteristics were examined by Student's *t* test.

Separately by the location, univariate and stepwise multiple linear regression analyses were used to study the relationships between birth and placental weights and maternal characteristics including parity, gestation at birth, maternal weights at 17, 26 and 34 weeks and post-delivery, and at 17 and 34 weeks of water weight, dry mass, ratio of water to weight as a percentage, and water and dry mass increase over this time. For the stepwise approach, a significance level of 0⋅25 was set as the criterion for entry of a predictor into the model and a level of 0⋅05 for a predictor to stay in the model. The determination of two predictors of birth weight by multiple regression to find a medium effect size (*f*^2^) of 0⋅15 and achieve a statistical power (1–*β*) of 0⋅90 and an *α* value of 0⋅05 required a total sample size of 88 (G*power Version 3.1.9.6, Kiel University, Germany). Pearson correlations, paired *t*-tests and Bland and Altman plots^(16)^ were used to compare the two methodologies for TBW determination. Statistical analyses were performed using Statistical Package for Social Sciences (SPSS) for Windows (version 25.0, IBM Corp.).

## Results

Of the 127 women who consented to consume total body water measured by the deuterium dilution technique (TBW-D_2_O) at 17 weeks, 109 were also measured at 34 weeks and ninety-nine of those had the weight of their child (fifty-one male and forty-eight female) at birth recorded. The weight of the placenta was measured for eighty-three of these women. Maternal weight at 17 weeks was not different when 127, 109 or 99 women were compared, overall or by location. Thirteen rural women and fifteen urban did not complete the measurements.

Almost half, 51 (52 %) lived in the rural area and 48 (48 %) in the urban area; 67 women (68 %) were nulliparous, twenty-eight had one and four had two previous pregnancies. Birth occurred at 39⋅3 ± 1⋅3 weeks of gestation. Overall, women were 154⋅8 ± 5⋅3 cm tall and, at 17 weeks, weighed 48⋅0 ± 6⋅9 kg (Table 1), with the mean BMI value of 20⋅2 ± 2⋅8 kg/m^2^. Women were educated for a median of 11 years and were of middle-class socio-economic status; the average standard of living index (SLI)^(17)^ was 37 ± 8, with a range of 13–61. The Indian government classifies an SLI of 0–14 as low, 15–24 as medium and 25–67 as high. Rural women were 3 years younger, weighed 5 kg less, gained 1⋅6 kg less weight between 17 and 34 weeks and had lighter placentas than urban but blood pressure, and the mean birth weight was not different between rural and urban (Table 1). The incidence of low birth weight (LBW <2⋅5 kg) in the rural environment was 21⋅6 % (11/51 births) and in the urban environment 10⋅4 % (5/48 births *P* for difference = 0⋅132). Crown-heel length measurements at birth were only available for forty-eight rural and forty-two urban births. Rural babies (47⋅8 ± 1⋅8 cm) compared with urban (49⋅2 ± 1⋅6 cm) were 1⋅3 cm shorter (*P* < 0⋅001).

**Table 1.** Characteristics of mothers at 17, 26 and 34 weeks and infant and placental birth weight overall and by the geographic location

Maternal characteristics	Overall (*n* 99)	Rural (*n* 51)	Urban (*n* 48)	Difference (95 % CI)	*P*
Age, years	23⋅2 ± 3⋅9	21⋅8 ± 3⋅1	24⋅6 ± 4⋅2	−2⋅7 (−4⋅2, −1⋅3)	<0⋅0001
Height, cm	154⋅8 ± 5⋅3	153⋅4 ± 5⋅2	156⋅2 ± 5⋅1	−2⋅8 (−4⋅9, −0⋅8)	0⋅008
Weight, kg					
17 weeks	48⋅0 ± 6⋅9	45⋅5 ± 4⋅8	50⋅7 ± 7⋅8	−5⋅2 (−7⋅8, −2⋅7)	<0⋅0001
26 weeks	52⋅7 ± 7⋅1	50⋅0 ± 4⋅9	55⋅6 ± 7⋅9	−5⋅7 (−8⋅3, −3⋅0)	<0⋅0001
34 weeks	54⋅6 ± 7⋅6	51⋅3 ± 5⋅3	58⋅1 ± 8⋅2	−6⋅9 (−9⋅6, −4⋅1)	<0⋅0001
Post-delivery	51⋅9 ± 8⋅2	47⋅8 ± 5⋅8	56⋅1 ± 8⋅2	−8⋅4 (−11⋅4, −5⋅3)	<0⋅0001
Weight gain, 17–34 weeks	6⋅7 ± 2⋅7	6⋅0 ± 2⋅2	7⋅5 ± 2⋅8	−1⋅6 (−2⋅6, −0⋅5)	0⋅003
TBW by D_2_O, kg					
17 weeks	25⋅5 ± 2⋅9	24⋅9 ± 2⋅9	26⋅1 ± 2⋅9	−1⋅2 (−2⋅3, −0⋅0)	0⋅046
34 weeks	29⋅8 ± 3⋅5	28⋅9 ± 3⋅2	30⋅8 ± 3⋅6	−1⋅9 (−3⋅3, −0⋅6)	0⋅006
TBW gain, 17–34 weeks, kg	4⋅4 ± 2⋅2	4⋅0 ± 2⋅4	4⋅8 ± 2⋅8	−0⋅8 (−1⋅6, 0⋅1)	0⋅092
TBW by BIS, kg					
17 weeks	23⋅6 ± 2⋅2	23⋅1 ± 1⋅9	24⋅2 ± 2⋅4	−1⋅0 (−1⋅9, −0⋅2)	0⋅022
26 weeks	25⋅2 ± 2⋅4	24⋅5 ± 1⋅9	25⋅8 ± 2⋅6	−1⋅3 (−2⋅2, −0⋅3)	0⋅007
34 weeks	26⋅0 ± 2⋅6	25⋅3 ± 2⋅0	26⋅8 ± 2⋅9	−1⋅5 (−2⋅5, −0⋅5)	0⋅003
Maternal dry weight, kg					
17 weeks	22⋅6 ± 5⋅0	20⋅6 ± 3⋅4	24⋅7 ± 5⋅7	−4⋅1 (−5⋅9, −2⋅2)	<0⋅0001
34 weeks	24⋅8 ± 5⋅4	22⋅4 ± 3⋅9	27⋅3 ± 5⋅6	−4⋅9 (−6⋅9, −3⋅0)	<0⋅0001
Dry weight gain, 17–34 weeks	2⋅2 ± 2⋅9	1⋅8 ± 3⋅0	2⋅6 ± 2⋅9	−0⋅9 (−2⋅0, 0⋅3)	0⋅151
TBW by D_2_O/weight, %					
17 weeks	53⋅4 ± 4⋅5	54⋅8 ± 4⋅4	51⋅8 ± 4⋅1	3⋅0 (1⋅3, 4⋅7)	0⋅001
34 weeks	55⋅0 ± 4⋅9	56⋅5 ± 4⋅9	53⋅3 ± 4⋅4	3⋅2 (1⋅3, 5⋅0)	0⋅001
TBW gain/weight gain, 17–34 weeks, %	72⋅0 ± 47⋅1	74⋅7 ± 58⋅0	69⋅2 ± 32⋅4	5⋅5 (−13⋅3, 24⋅4)	0⋅563
Systolic BP, mmHg					
17 weeks	103 ± 7	107 ± 7	101 ± 8	3 (−0, 5)	0⋅075
26 weeks	100 ± 8	100 ± 7	99 ± 9	1 (−2, 4)	0⋅448
34 weeks	104 ± 10	104 ± 8	104 ± 11	−0 (−4, 4)	0⋅905
Diastolic BP, mmHg					
17 weeks	59 ± 5	59 ± 5	59 ± 6	−0 (−2, 2)	0⋅892
26 weeks	60 ± 6	59 ± 6	61 ± 5	−2 (−5, −0)	0⋅040
34 weeks	63 ± 7	62 ± 8	64 ± 7	−2 (−4, 1)	0⋅294
Birth weight, g	2783 ± 344	2720 ± 358	2849 ± 318	−128 (68, −263)	0⋅063
Gestation at birth, weeks	39⋅3 ± 1⋅3	39⋅4 ± 1⋅3	39⋅2 ± 1⋅3	0⋅2 (−0⋅3, 0⋅8)	0⋅369
Placental weight, g (*n* 83)	440 ± 96 *n* 83	404 ± 73 *n* 44	481 ± 102 *n* 39	−77 (19, −116)	<0⋅0001

BIS, bioimpedance spectroscopy; BP, blood pressure; D_2_O, deuterated water; TBW, total body water.

At 17 and 34 weeks, rural women had a higher proportion of water to total weight implying lower fatness. The ratio of the mass of water gained to total mass gained between 17 and 34 weeks was not different by the location (Table 1). Systolic and diastolic blood pressures did not change from 17 to 34 weeks. Placental weight was associated with birth weight in both rural (*r*  0⋅438, *P* = 0⋅003) and urban women (*r*  0⋅624, *P* < 0⋅001).

On multivariate analysis, the birth weight of babies of both rural and urban women was positively and independently associated with parity and gestation at birth (Tables 2 and 3). For rural women, a unit increase in parity was associated with a 238 g increase in birth weight and a week of gestation 70 g higher birth weight. These respective increases in birth weight for urban women were 113 and 235 g. Only for rural women was an increase in dry mass independently associated with a higher birth weight. Also, only for rural women, univariate analysis showed that a *decrease* in percentage body water as a percentage of body weight from 17 to 34 weeks was associated with a higher birth weight. This implies proportionally more body fat gain – as fat is a component of dry mass which is associated with a higher birth weight.

**Table 2.** Associations between characteristics of fifty-one rural mothers during pregnancy and birth weight (g) by univariate and multiple regression analyses

Maternal characteristic	Univariate			Multivariate		
	*β*	95 % CI	*P*	*β*	95 % CI	*P*
Infant sex (male = 0, female = 1)	43⋅4	−160⋅2, 246⋅9	0⋅670			
Gestation at birth, weeks	**89⋅9**	**16⋅0, 163⋅8**	**0**⋅**018**	**237**⋅**6**	**96⋅8, 378⋅4**	**0**⋅**001**
Parity	**204**⋅**3**	**45⋅8, 362⋅8**	**0**⋅**013**	**70**⋅**4**	**4⋅7, 136⋅0**	**0**⋅**036**
Height, cm	0⋅54	–19⋅10, 20⋅17	0⋅956			
Weight, kg						
17 weeks	3⋅58	−17⋅86, 25⋅01	0⋅739			
26 weeks	8⋅269	−12⋅46, 29⋅00	0⋅427			
34 weeks	11⋅23	−8⋅05, 30⋅52	0⋅248			
Post-delivery	15⋅37	−3⋅14, 33⋅88	0⋅101			
Weight gain, 17–34 weeks	35⋅58	−9⋅07, 80⋅22	0⋅116			
Total body water, kg by D_2_O						
17 weeks	15⋅04	−20⋅54, 50⋅62	0⋅400			
34 weeks	−2⋅70	−35⋅11, 29⋅71	0⋅868			
Water gain, 17–34 weeks, kg	−26⋅39	−68⋅70, 15⋅93	0⋅216			
Maternal dry weight, kg						
17 weeks	−3⋅69	−34⋅21, 26⋅8	0⋅809			
34 weeks	22⋅31	−3⋅33, 47⋅96	0⋅087			
Dry weight gain, 17–34 weeks	**41**⋅**83**	**9⋅87, 73⋅79**	**0**⋅**011**	**37**⋅**6**	**7⋅4, 67⋅7**	**0**⋅**016**
% Water/total weight						
Water/total weight %, 17 weeks	10⋅79	−12⋅36, 33⋅94	0⋅354			
Water/total weight %, 34 weeks	−6⋅21	−36⋅77, 4⋅33	0⋅119			
Water/weight gain %, 17–34 weeks	**−1**⋅**93**	**−3⋅59, −0⋅216**	**0**⋅**028**			

Bold text indicates statistical significance with a *P* value <0·05.

**Table 3.** Associations between characteristics of forty-eight urban mothers during pregnancy and birth weight (g) by univariate and multiple regression analyses

Maternal characteristic	Univariate			Multivariate		
	*β*	95 % CI	*P*	*β*	95 % CI	*P*
Infant sex male = 0, female = 1	−88⋅5	−273⋅7, 96⋅8	0⋅341			
Gestation at birth, weeks	**102⋅2**	**34⋅6, 169⋅8**	**0**⋅**004**	**112**⋅**5**	**48⋅3, 176⋅8**	**0**⋅**001**
Parity	178⋅7	−1⋅3,358⋅8	0⋅052	**235**⋅**1**	**70⋅5, 400⋅0**	**0**⋅**006**
Height, cm	10⋅94	−7⋅42, 29⋅30	0⋅237			
Weight, kg						
17 weeks	2⋅13	−9⋅90, 14⋅16	0⋅724			
26 weeks	4⋅73	−7⋅23, 16⋅69	0⋅423			
34 weeks	4⋅07	−7⋅44, 15⋅59	0⋅480			
Post-delivery	5⋅69	−6⋅47, 17⋅84	0⋅350			
Weight gain, 17–34 weeks	16⋅65	−16⋅21, 49⋅51	0⋅313			
Total body water, kg by D_2_O						
17 weeks	2⋅47	−30⋅67, 35⋅61	0⋅881			
34 weeks	6⋅39	−19⋅56, 32⋅34	0⋅622			
Water gain, 17–34 weeks, kg	16⋅65	−31⋅20, 64⋅50	0⋅487			
Maternal dry weight, kg						
17 weeks	3⋅44	−13⋅18, 20⋅07	0⋅679			
34 weeks	5⋅88	−10⋅78, 22⋅53	0⋅481			
Dry weight gain, 17–34 weeks	9⋅13	−23⋅44, 41⋅70	0⋅575			
% Water/total weight						
Water/total weight %, 17 weeks	−3⋅32	−26⋅30, 19⋅67	0⋅773			
Water/total weight %, 34 weeks	−4⋅50	−25⋅71, 16⋅71	0⋅671			
Water/weight gain %, 17–34 weeks	0⋅64	−2⋅28, 3⋅56	0⋅661			

Bold text indicates statistical significance with a *P* value <0·05.

The only statistically significant determinant of placental weight was the number of weeks of gestation at birth for rural (*β* 20⋅0; 95 % CI 3⋅1, 36⋅8; *P* = 0⋅021), but not for urban, women.

**Fig. 1.**
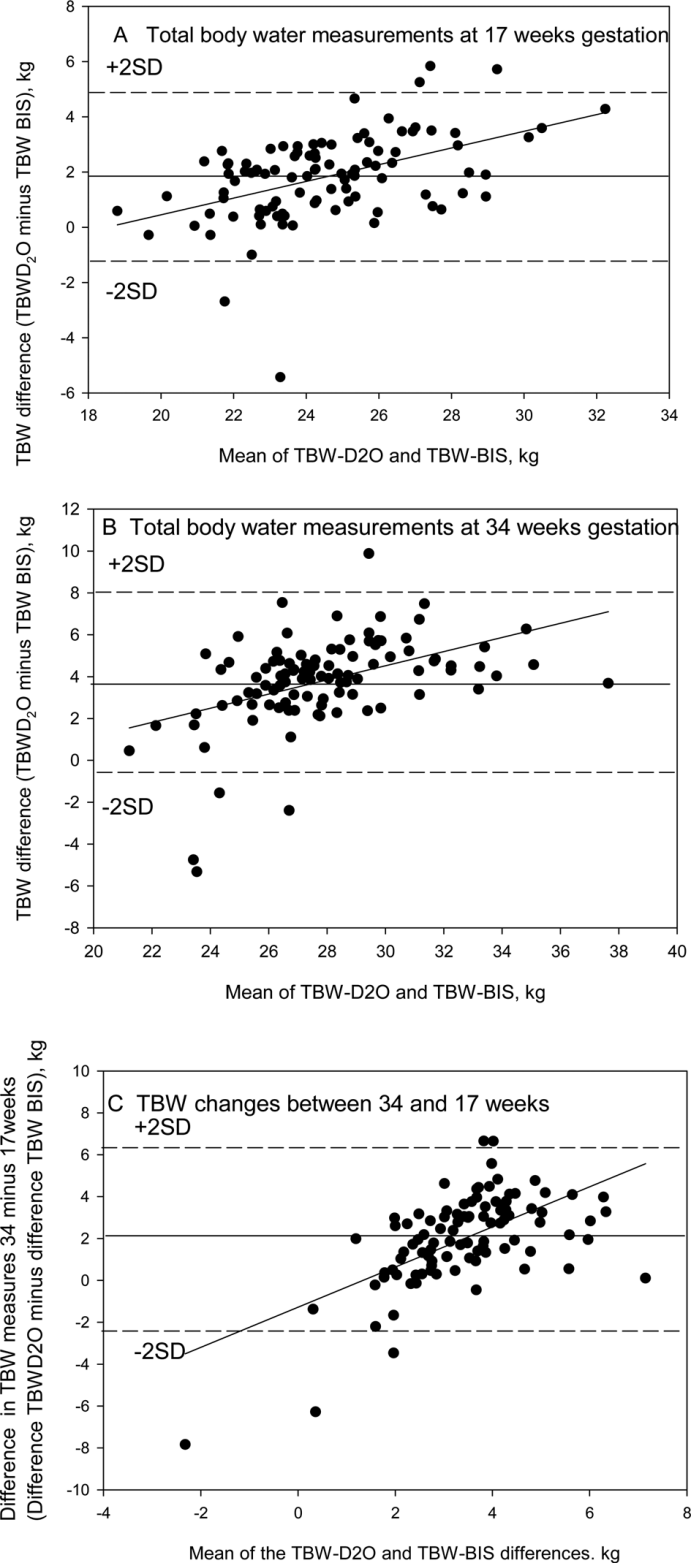
Comparison at 17 (a) and 34 weeks (b) of pregnancy of the difference between total body water (TBW) as estimated by deuterium dioxide dilution (TBW D_2_O) and TBW as assessed by whole-body bioimpedance spectroscopy (TBW-BIS) with the mean of the two methods. In (c), the differences between the TBW changes over these two time points as assessed by the two methods are compared against the mean difference. Solid horizontal lines indicate the means of the differences and dashed lines the limits of agreement (mean difference ± 2 sd).

The correlations between BIS and D_2_O estimates of TBW were 0⋅849 (*P* < 0⋅001) at 17 weeks and 0⋅790 (*P* < 0⋅001) at 34 weeks. However, we found that on average BIS underestimated the D_2_O measured water space (Fig. 1) and the underestimation increased with the size of that compartment at both weeks 17 (*r* 0⋅475, *P* < 0⋅001, Fig. 1(a)) and 34 (*r* 0⋅452, *P* < 0⋅001 Fig. 1(b)). For the changes in TBW between 17 and 34 weeks, the correlation between these two methods of estimation was 0⋅281 (*P* = 0⋅005). The Bland and Altman plot (Fig. 1(c)) indicated an average underestimation of the change measured by BIS compared to D_2_O but with some overestimation at lower levels of change and, predominantly, underestimation at higher levels of change (*r*  0⋅604, *P* < 0⋅001).

## Discussion

This investigation of maternal total body water at 17 and 34 weeks found that only for rural women but not for urban, a relative increase in dry mass or a *decrease* in percentage body water between 17 and 34 weeks was associated with a higher birth weight. This implies proportionally more body fat gain, a component of dry mass, was associated with a higher birth weight. Since 17–34 weeks encompass trimester 2 and 6 weeks of trimester 3, we cannot compare the present results directly to studies that measured body composition within a trimester. Other researchers^(18)^ have found that a higher rate of GWG in trimester 2 was associated with higher birth weight and higher GWG in both trimester 2 and 3 with larger gains in maternal fat mass measured by underwater weighing.

The women in our study would not be considered underweight, although compared to European and Asian Indian women in developed countries, e.g. New Zealand^(19)^, total body mass was low and the height was short. The utility of BMI cutpoints is recognised as different for Asian populations compared to European,^(20)^ but there are no specific BMI cutpoints for pregnant Asian women.

In comparison with the Institute of Medicine (IOM) guidelines for GWG for women with a BMI of 20 kg/m^2^, 11⋅5–16 kg is recommended,^(2)^ but the mean weight gain in the present study of 6⋅7 kg was considerably less, albeit that the gain for our women was between weeks 17 and 34. A further 6 weeks of pregnancy could result in a further 3 kg of body weight. In addition, mean birth weight was 2⋅8 kg, which is not considered low by the IOM, but is less than the 3⋅4 kg reported by Butte *et al.*^(21)^ for a water gain of 7⋅1 kg.

The rate of recommended weight gain/week in the second and third trimester is ~0⋅4 kg/week,^(2)^ which adds to an expected 0⋅5–2 kg gain in the first trimester. Weight gain over the 17 weeks from 17 to 34 weeks was, on average, 0⋅4 kg/week, which is within the IOM guideline.

Lederman *et al.* studied TBW in 200 women in New York (weight at week 14, 65 kg) in early and late pregnancy and reported that, at term, maternal TBW, not fat, was significantly associated with infant birth weight^(22)^. For each 1 litre increase in body water at week 37, birth weight was increased by 35 g. In Chile, Mardones-Santander *et al.* measured TBW by D_2_O in 224 pregnant women at term and found that fat-free mass was the most important maternal body component associated with birth weight^(23)^. One kg of additional fat-free mass was associated with 20 g of extra birth weight and 1 kg of additional fat mass with 18 g; a 1 week increase in gestational age at delivery was associated with 79 g of extra birth weight. However, the estimate of fat-free mass at term assumed a hydration of 75 % as modelled by van Raaij *et al.*^(24)^. Butte *et al.* assessed body composition, including TBW by deuterium dilution, using the four-compartment model in sixty-three women of low, normal and high BMI, from before pregnancy, at 9, 22 and 36 weeks of pregnancy and 2, 6 and 27 weeks of postpartum^(21)^. Women with higher BMI gained more weight and fat mass in pregnancy, but all had similar gains in TBW, protein and fat-free mass. Birth weight of offspring was significantly correlated with maternal pre-pregnancy weight, pre-pregnancy fat mass and gain in maternal weight, TBW and fat-free mass, but not with gain in fat mass^(21)^. This is in agreement with our findings that water and dry mass gained by urban and rural women were not statistically different, but weight gain was higher for urban, suggesting that more fat mass was gained by urban women. Possible explanations include the level of physical activity and dietary patterns as the rural women were most often engaged in agriculture.

In agreement with BIS results at 32 weeks of gestation reported by Lof and Forsum^(25)^, where tracer dilution was also used as a reference TBW measure, we report that in pregnancy at 17 and 34 weeks, BIS underestimated total body water. The mean difference in both studies was 3⋅8 kg, and the extent of underestimation increased with weeks of gestation. Both studies used Cole model-fitting and Hanai mixture theory to estimate TBW by BIS. A number of factors may contribute to the TBW underestimation by BIS. During pregnancy, a substantial fraction of the increased TBW will be sequestered in the trunk, which contributes very little to the measured resistance of the whole body that is predominantly from the legs and arms with their much smaller cross-sectional areas. The increased underestimation observed from 17 to 34 weeks is supported by this explanation. Secondly, the shape factor used in the BIS formulae, based on the lengths and circumferences of the limbs and the trunk, will be altered during pregnancy^(26)^. Thirdly, the resistivity coefficients adopted by the manufacturer's software may not apply in pregnancy, given that electrolyte concentrations change during pregnancy. We observed significant linear relationships between the average TBW estimates from dilution and BIS and the differences between the two techniques which were not seen in the Lof and Forsum study^(25)^. Refinements to the BIS model for application in late pregnancy may improve accuracy and precision and permit the appropriate evaluation of the utility of TBW estimates by this approach in individual women.

This study was limited by its relatively small sample size but strengthened by the sampling of women living in both rural and urban areas, the comprehensive and diligent longitudinal measurements and the validity of measurements of body water by D_2_O. This investigation adds to a paucity of literature concerning body composition and GWG of Indian women and highlights differences in body composition during pregnancy related to rural and urban environments and possible impacts of the nutrition transition on body composition in pregnancy and fetal growth.
